# Extracellular Vesicles in Viral Replication and Pathogenesis and Their Potential Role in Therapeutic Intervention

**DOI:** 10.3390/v12080887

**Published:** 2020-08-13

**Authors:** Asit Kumar, Sunitha Kodidela, Erene Tadrous, Theodore James Cory, Crystal Martin Walker, Amber Marie Smith, Ahona Mukherjee, Santosh Kumar

**Affiliations:** 1 Department of Pharmaceutical Sciences, University of Tennessee Health Science Center, Memphis, TN 38163, USA; skodidel@uthsc.edu (S.K.); etadrous@uthsc.edu (E.T.); imahona@gmail.com (A.M.); 2 Department of Clinical Pharmacy and Translational Science, College of Pharmacy, University of Tennessee Health Science Center, Memphis, TN 38163, USA; tcory1@uthsc.edu; 3 College of Nursing, University of Tennessee Health Science Center, Memphis, TN 38163, USA; cmarti47@uthsc.edu; 4 Department of Pediatrics, University of Tennessee Health Science Center, Memphis, TN 38163, USA; amber.smith@uthsc.edu

**Keywords:** extracellular vesicles, exosomes, microvesicles/microparticles, viruses, infection, antiviral and antiretroviral drug, immune response

## Abstract

Extracellular vesicles (EVs) have shown their potential as a carrier of molecular information, and they have been involved in physiological functions and diseases caused by viral infections. Virus-infected cells secrete various lipid-bound vesicles, including endosome pathway-derived exosomes and microvesicles/microparticles that are released from the plasma membrane. They are released via a direct outward budding and fission of plasma membrane blebs into the extracellular space to either facilitate virus propagation or regulate the immune responses. Moreover, EVs generated by virus-infected cells can incorporate virulence factors including viral protein and viral genetic material, and thus can resemble noninfectious viruses. Interactions of EVs with recipient cells have been shown to activate signaling pathways that may contribute to a sustained cellular response towards viral infections. EVs, by utilizing a complex set of cargos, can play a regulatory role in viral infection, both by facilitating and suppressing the infection. EV-based antiviral and antiretroviral drug delivery approaches provide an opportunity for targeted drug delivery. In this review, we summarize the literature on EVs, their associated involvement in transmission in viral infections, and potential therapeutic implications.

## 1. Introduction

Cells mediate intercellular communication and modulation of immune responses through shedding and release of extracellular vesicles (EVs) [1]. These EVs are diverse and originate from plasma membrane and endosomes and include exosomes, micro-vesicles (MVs, also known as microparticles), and apoptotic bodies. They are categorized based on their biogenesis, release pathways, size, content, and function [2]. EVs shed from plasma membranes are generally referred to as MVs [3,4,5], while vesicles that are generated by inward budding of endosomes to form multivesicular bodies (MVBs) that fuse with the plasma membrane, and release into the extracellular environment, are known as exosomes [6,7]; whereas, cells undergoing apoptosis can release vesicles or cell filaments exclusively from the plasma membrane, called apoptotic bodies [8,9]. Depending on their biogenesis pathway and cellular origin, EVs can be packaged with functional proteins, lipids, mRNA/miRNA, and other cytosolic components. These EVs are either beneficial or detrimental to the host’s immune response during disease, injury, viral and pathogen infection [10,11,12,13,14,15]. It is now evident that viruses can use extracellular vesicles that can enhance viral propagation and spread. For instance, vesicles derived from apoptotic cells can help viral infections such as HIV by inhibiting dendritic cell activation and function [16]. EVs released by virus-infected cells contain specific cellular components and viral proteins and sometimes parts of viral genetic materials [17,18,19], many of which aid viral propagation and facilitate viral persistence during the hostile environment of the host’s immune response [20,21]. Recent advances in the molecular mechanisms of viral infection and pathogenesis yield a physiological link between EVs and viruses [22]. Viruses exploit EVs for fundamental cellular processes such as viral entry into host cells, evade the immune response, and spread viral proteins and genetic materials, including functional, noncoding microRNAs (miRNAs) [20,23,24]. Similar to viruses, EVs can bind a cellular membrane and enter target cells either through fusion or endocytosis, and trigger signaling and inflammatory responses in target cells [25,26,27]. Biological responses triggered by these target cells are initiated after receiving EVs that either carry host cell components, viral proteins, or fragments of the viral genome. In some cases, non-enveloped viruses such as hepatitis A virus (HAV) use alternative infection capabilities via EVs that provide an “envelope” to non-enveloped viruses [28]; whereas, in other cases, hepatitis C-infected cells release EVs containing whole viral genomes that generate new infectious viral particles in target cells [29]. EVs are not only involved in supporting viral infection but also able to elicit an immune response against viruses [30]. In this review, we emphasize the role of EVs in viral infection and pathogenesis. Moreover, we review the potential of EVs as therapeutic delivery agents for antiviral and antiretroviral drugs.

## 2. EV Biogenesis and Uptake

EVs are nanoscale membrane vesicles, which are actively released by cells. They are broadly classified into exosomes, microvesicles, and apoptotic bodies based on their origin, size, content, and corresponding markers [31]. Recent studies have suggested that both EVs and viruses, in particular retroviruses have a common biogenesis pathway, as well as structural and functional resemblance [32].

### 2.1. Exosomes

Exosomes are vesicles of endocytic origin and their size usually ranges from 30–120 nm [33]. Exosomal markers include tetraspanins (TSPAN29 and TSPAN30, ESCRT components, and TSG101). The invasion of the plasma membrane inwards forms the early endosome and the limiting membrane of the later endosome sprouts further to form the MVBs. MVBs are characterized by the invagination of the inner body membrane, which results in the formation of intraluminal vesicles (ILVs) [34]. During this process, cytoplasmic components and certain peripheral proteins are integrated into them. The ILVs accumulated in the MVB lumen have two routes. One is to diffuse with the lysosomes, which causes the contents of the vesicles to degrade, and the other is fusion with the cytoplasmic membrane and release of the vesicles to the extracellular space by exocytosis, referred as “exosomes” [35]. Loading of biological cargos into ILVs involves the endosomal sorting complexes required for transport (ESCRT) complexes (ESCRT-0, -I, -II, -III and the Vps4) and other accessory proteins such as Alix/PDCd6IP, TSG101, HRS, etc. [36,37].

In addition to ESCRT, other mechanisms can also produce exosomes of certain biochemical components. For instance, in some cells production of exosomes requires lipid ceramide and neutral sphingomyelinase [38], an enzyme that converts sphingomyelin to ceramide, and related proteins including phospholipase D2 that hydrolyzes phosphatidylcholine into phosphatidic acid and DGK alpha [39,40]. Another mechanism of exosome release relies on small GTPases such as Rab27a/b [41], Rab 7, 11, 31, and 35 in some cells, or soluble *N*-ethylmaleimide-sensitive factor attachment protein receptor (SNARE) family proteins like YKT6 [42,43], vesicle-associated membrane protein 7 (VAMP7) [44,45], CD9, and CD63. These proteins are involved in exosome biogenesis and are commonly used as markers of exosomes [46]. Several studies have demonstrated the effect of viral infections on the host exosomes as they altered primary cellular processes related to exosome biogenesis [47,48]. For instance, several viruses, in particular retroviruses, can enter the cells through endocytosis and hijack and use exosomal pathways for their replication and pathogenesis [49]. Viruses such as hepatitis C virus (HCV), West Nile virus (WNV), Zika virus (ZV), and Dengue virus (DENV) enter this pathway by clathrin-mediated or receptor-mediated endocytosis [50,51,52,53,54,55,56]. Another virus that can utilize the endosomal/exosomal system for its replication and pathogenesis is the human immunodeficiency virus (HIV). Exosomes and HIV particles have similarities in terms of biogenesis, biophysical/molecular properties, and cellular uptake mechanisms [57]. Based on these similarities between exosomes and HIV, the “Trojan exosome hypothesis” has been proposed. This suggests that HIV could exploit the exosome system to infect cells independently of envelope protein–receptor interactions [58,59]. However, this hypothesis was quickly disputed by others, suggesting that HIV proteins aggregate at the plasma membrane, and viruses are secreted by budding, not by interaction with the exosome secretion pathway [60,61,62,63,64].

### 2.2. Microvesicles

Microvesicles represent a relatively heterogeneous population of vesicles and their size ranges from 50–600 nm [65]. MVs are formed by outward germination and fission of cell membranes, which can be controlled by phospholipid redistribution and cytoskeletal protein contraction [66]. MVs formation is induced by translocation of phosphatidylserine to the outer membrane leaflet through the activity of amino-phospholipid translocases. ADP-ribosylation factor 6 (ARF6) causes budding of these MVs by initiating a signaling cascade that activates phospholipase D (PLD) [67,68]. PLD recruits extracellular signal-regulated kinase (ERK) to the plasma membrane leading to activation of myosin light-chain kinase (MLCK), which triggers the release of MVs [67,69]. MVs are involved in antigen presentation and transfer of the major histocompatibility complex (MHC) molecules and antigens, thereby participating in immune regulation [70,71] and involvement in other processes [39,72].

### 2.3. Apoptotic Bodies

Unlike exosomes and microvesicles, which are released during normal cellular processes, apoptotic bodies are formed only during programmed cell death [73,74]. Apoptotic bodies’ size ranges from 500–4000 nm. During apoptosis, the cell undergoes morphological changes and shrinks to a smaller size with densely packed cytoplasm and other organelles, and eventually their nucleus disintegrates [75]. Further, the cells form blebs on its surface and disintegrate into small fragments called apoptotic bodies. These are characterized by the presence of organelles within the vesicles [73] and are cleared from the body by phagocytosis by specific mechanisms [76,77]. The most commonly used identifiers of apoptotic bodies are Annexin V, thrombospondin, and C3b [78]. Limited knowledge exists in the literature regarding the role of apoptotic bodies in cell-cell communication during viral infection and their contribution to viral pathogenesis. To understand their possible role and function in intercellular communication, numerous in-depth studies are warranted in the future.

### 2.4. EV Uptake

Uptake of EV seems to depend on the type of recipient cell, its physiological state, and recognition of ligands or receptors on the recipient cell and EVs. Cells broadly internalize EVs either by fusion with the plasma membrane or via endocytosis. Internalization of EVs by recipient cells occurs by various mechanisms of endocytosis including clathrin-dependent and clathrin-independent mechanisms such as caveolin-mediated uptake, macro-pinocytosis, phagocytosis, and lipid raft-mediated internalization [79,80]. EV uptake is an energy-dependent process [79]. Neurons internalize oligodendrocyte-derived exosomes by clathrin-mediated endocytosis [81], whereas microglia internalize exosomes by micropinocytosis [82]. Epithelial cells internalize exosomes by caveola-dependent endocytosis [83], while dendritic cells internalize EVs through lipid raft domains [84]. Different methods are employed to detect EV uptake, among which the most used method is the use of fluorescent lipid membrane dyes to stain EV membranes. Examples of such dyes include PKH67, PKH26, rhodamine B, DiI, and DiD [79,80,85,86]. The internalization of EVs by recipient cells can be measured using methods such as flow cytometry and confocal microscopy [86,87].

## 3. EV Isolation Method and Characterization Techniques

EVs and viruses are highly heterogeneous in size, structure, and biogenesis, and therefore they cause apparent difficulties in distinguishing and separating EVs from viruses. Even though EVs and viruses overlap in size and biophysical properties, EVs far outnumber high-titer viruses during infection [88].

In the past decade, a multitude of isolation and purification methods for EVs and virus particles have been developed. Differential centrifugation/ultracentrifugation (UC) technique is widely used for the isolation of EVs from cell cultures’ media and biological fluids that contain viruses [89]. Although this technique is considered as the gold standard of EV isolation, it often coprecipitates with proteins and lipoproteins that can affect sample purity and may interfere with downstream analysis [90,91], limiting its use in hospital settings. This limitation can be overcome by including multiple isolations and characterization techniques such as antibody-based immunoaffinity purification, tangential flow filtration (TFF), and nano-flow cytometry (nFCM) [92,93,94,95]. However, each of these methods has its limitations, which need to be considered before planning EV isolation and purification. For instance, EV isolation using the antibody-based immunoaffinity purification method provides a refined EV population but is limited by the sample volume and amount of final product [96]. Moreover, the expression level of EV markers such as CD9, CD63, and CD81 can vary depending on the EV origin and physiological condition, requiring a combination of markers to be used. Compared to UC, the TFF method can be effective in obtaining EV-enriched formulations from a large volume of samples. However, TFF is likely to cost higher than conventional EV isolation methods. Further studies are required to explore the utilization of TFF for clinical studies [92]. Due to limitations associated with isolation procedures, and lack of a standardized isolation process, a validated good manufacturing practice (GMP)-compliant procedure is desperately needed. Bari et al. employed conditioned media from mesenchymal stem/stromal cells for the secretome/EV isolation. A key aspect of their study is a large-scale secretome or EV isolation process using UC and TFF that complies with GMP, which allows standardized and pharmaceutical grade products suitable for clinical applications [97,98]. The use of nFCM is reported as a new benchmark for quality assessment of EVs. Phenotyping of single particles is possible through nFCM using immunofluorescent labeling of EVs [94]. However, the limitations in resolution and detection varied depending on the criteria used to define the EV populations based on markers [99] that have excluded many researchers widely utilizing this technique. Besides, an nFCM based method can be challenging to develop and to validate EV characterization, given the specific EV population measurement and due to the lack of standard guidelines for handling and analyzing a variety of samples with appropriate normative controls in nFCM. Li K et al. have developed an approach termed Cushioned–Density Gradient Ultracentrifugation (C-DGUC), a variant of ultracentrifugation, for EV refinement [100]. In this approach, samples were processed through a density gradient cushion such as iodixanol (Optiprep™) and centrifugal force to maximizes EV recovery followed by density gradient ultracentrifugation steps that eventually provide high-purity purification of EVs by effectively removing protein aggregates. However, EVs can lose integrity while isolated from a fixed density range [101]. Polyethylene glycol (PEG) precipitation followed by iodixanol density separation has recently become a useful method to pull down EVs, viruses, and proteins or protein-RNA aggregates within a sample, followed by an additional centrifugation step. This method results in a significantly higher yield of EVs in comparison to the conventional UC method [102]. The contents of EVs vary greatly depending upon the condition of the parent cell. Thus, apart from characterizing the vesicles, identifying these contents reveals a breadth of information regarding the parent cells. The International Society for Extracellular Vesicles (ISEV) 2018 guidelines should be followed when isolating EVs from cells or plasma/biological fluids for drug encapsulation. The most pragmatic approach appears to be the isolation of EVs using a commercial kit and size exclusion chromatography (SEC; also known as gel filtration) methods followed by microfiltration of samples using filters with pore diameters of 0.1, 0.22, or 0.45 μm depending on the size of vesicles required. In SEC, EVs are separated from other material according to differences in sizes (hydrodynamic radii) [103] that gives this technique the upper edge over conventional methods and can be effectively used for a variety of complex biological samples such as body fluid, blood/plasma, urine, and breast milk [104,105,106,107]. Isolation of high-purity EVs from samples containing virions is challenging since both EVs and some viruses, in this case, retroviruses, are similar in size. As of now, no validated protocol is available to specifically separate EVs from virions that are similar in size and carry the same markers [32]. However, a study has demonstrated that defective viruses could be separated from naturally occurring viruses based on differences in buoyant densities [108]. EVs loaded with drugs to treat viral diseases require them to target majorly infected cells or tissues. When considering EVs as personalized therapeutic carriers, surface engineering of EVs is required that can be performed using covalent and noncovalent modification [109,110,111]. It is important to optimize the method of isolation for EVs for drug loading on a case-to-case basis. Upon loading drugs to these EVs, the EVs can be further separated using a sucrose gradient that utilizes iodixanol and characters each fraction for loading efficiency and total loading. The EV fractions with optimally loaded drugs can be further characterized by their size, shape, and marker proteins for further use. 

## 4. EVs in Viral Transmission and Pathogenesis: A Brief Overview 

EVs released by virus-infected cells can incorporate protein molecules, derived from viral genes involved in viral assembly. Delivery of the EV-associated virulence molecules affects recipient cells by rendering them particularly vulnerable to viral infection (Table 1). Moreover, incorporating viral proteins can trigger cell death of non-participating immune cells [112] that would contribute to the heavy loss of immune cells during the early stages of viral infection or low viral load. Intercellular transfer of viral proteins and viral cell surface receptors by EVs not only facilitates evasion of the host’s immune response by suppressing antibody production in lymphocytes but also makes immune cells susceptible to viral infection [32,113]. However, while evidence indicates that EVs can, directly and indirectly, mediate the antiviral response, their role in regulating immune response is not yet fully elucidated in vivo.

### 4.1. HIV and EVs 

In HIV, EVs are thought to play an important role in disease progression through multiple mechanisms. Viral components may be packaged in EVs, which can then be delivered to uninfected cells, modulating the systemic inflammatory status. For instance, HIV-infected cell-derived exosomes carry viral protein Nef that induces apoptosis in immune cells and reduces the blood–brain barrier (BBB) integrity to spread viral infection in the brain [112,114]. It has been shown that EVs released during HIV infection are heterogeneous including size variability. A study has shown that treatment-naïve people living with HIV/AIDS (PLWHA) contain EVs larger in size and numbers compared to PLWHA who were either virally suppressed, elite controllers, or healthy controls [146]. Additionally, CD4 counts and the abundance of EVs in the blood were inversely correlated, with low CD4 counts associated with more abundant EVs. Interestingly, there was no relationship between CD4 counts and EV size. Both size and abundance were also inversely correlated with neutrophils and platelet counts, as well as the CD4/CD8 ratio, all of which are markers of disease progression [146]. This suggests that EVs may function as a biomarker for HIV disease progression.

Other studies have observed similar findings. In cells treated with antiretroviral drugs (ARVs), increases in relative EV production has been observed [102], along with decreased loading of genomic, but not non-coding, RNA into EVs from cells, which were treated with ARVs, as opposed to untreated cells. Additionally, treatment with interferon-alpha increased the packaging of viral RNA into EVs. The authors suggest that this occurs because ARV or interferon prevents the release of viral particles from cells, which then allows for viral RNA to be packaged into EVs due to the increased presence of viral RNA in the cell. In addition to viral RNA, a variety of molecules, e.g., viral & host proteins, cellular markers, miRNA, inflammatory molecules such as oxidative stress markers, chemokines and cytokines can also be packaged into EVs [20,115,116,117,119,121]. A study showed that the viral envelop (Env) protein can be packaged into EVs from infected cells [147]. The Env-containing EVs can increase susceptibility to viral infection in cell culture experiments, and depletion of Env-containing EVs showed decreased susceptibility to viral infection. 

Altered levels of proteins in plasma EVs are often described upon viral infection. For example, various examples of significantly altered expression of proteins, and markers associated with cellular stress, have been reported in plasma EVs derived from HIV and HTLV-1 infected patients. However, the mechanism of specific packaging of these proteins and markers in EVs and their role in intercellular communication was not elucidated [148,149]. Blood plasma can be considered as disease biomarkers since it contains glycoproteins and cellular markers carried in EVs [150]. Dysregulation of cytokines and chemokines is often associated with HIV infection and subsequently contribute to the viral pathogenesis [20,151,152]. Moreover, the use of substances such as alcohol, tobacco, and drugs is prevalent among HIV-infected individuals [153,154,155,156]. Circulating inflammatory cytokines have been found to be elevated in HIV-positive substance users [117,151,157,158]. In prior studies, we demonstrated that exosomes derived from HIV-infected monocytes/macrophage cells exert a protective effect against cytotoxicity and viral replication in HIV-infected macrophages. 

However, exosomes derived from HIV-infected cells lost their protective capacity that could be due to the selective packaging of cytochrome P450 (CYPs) and antioxidant enzyme (AOE) mRNAs in exosomes [21]. Similar to the previous study, exposure to cigarette smoke condensate (CSC) increased the packaging of cytokines, especially IL-6 and CYPs (1A1 and 1B1) in EVs isolated from HIV-infected U1 macrophages [116]. Conversely, EV packaging of AOEs (SOD-1 and catalase) decreased in HIV-infected U1 macrophages more than in uninfected U937 macrophages [116]. Recently, our group showed that the astrocytic and neuronal-specific markers (GFAP and L1CAM) can be packaged in EVs and circulate in plasma, which is further elevated in the presence of HIV infection, alcohol, and/or tobacco [121]. Human cytidine deaminase APOBEC3G (A3G) can be packaged in EVs and inhibit HIV replication with its potential DNA-editing activity [118].

### 4.2. HPV and EVs 

HPV-infected cells release EVs that make other cells more susceptible to infection as they deliver proteins that affect viral expression, and subsequently tumor development [19,122,159]. To enhance protein delivery and HPV replication, HPV-infected cells hijack EV signaling pathways to control the quantitative and qualitative release of EVs from HPV-infected cells [123,159,160,161]. As tumor genes and proteins are persistently expressed from EVs, this contributes to HPV cancer cell growth [122], thereby making the signaling pathways of EVs harmful to the host. The oxidative stress released from HPV-infected cells into EVs should also be considered detrimental to the host as this stress has the potential to induce viral replication of other viruses such as HIV-1 [120]. To make matters more complex, the signaling pathways of EVs are not limited to increased HPV replication as the release of EVs can also promote an adaptive immune response that becomes beneficial to the host [30]. For example, in the setting of HPV replication and tumor progression, EVs have prompted immune activation in head and neck cancers and are being considered as biomarkers for improved clinical outcomes [162,163,164,165]. Besides, endogenously engineered EVs are being considered as a novel method to deliver anti-HPV immunotherapy [166], thus making them yet another way to improve clinical outcomes. Unique miRNA signatures were found in EVs released from cervical cancer affected cells that were associated with HPV status [124,125,126,127,167]. 

### 4.3. Influenza Virus and EVs 

During influenza virus infection, EVs carrying host miRNA or viral epitopes are thought to be integral to antigen transfer, reducing virus spread, and immune regulation [168]. For example, influenza virus hemagglutinin (HA) epitopes enclosed within exosomes on MHCII molecules have been shown to improve the efficiency of antigen delivery to immune cells [169]. Further, exosomal-like vesicles carrying mucin molecules such as MUC1, MUC4, and MUC16 can bind sialic acids and neutralize influenza viruses [128], which may help reduce virus dissemination. Virus replication can also be blocked by some highly upregulated exosomal miRNAs, such as the type I interferon-inducing hsa-miR-1975 and miR-483-3p [129,130]. Also, these EVs excite other proinflammatory cytokines, such as IL-6, TNF-α, and IFN-β [129,170], although their efficacy may be dependent on cell source, maturity, and MHC molecules. Macrophages have been shown to produce thousands of proteins within exosomal vesicles in response to influenza infection. These EVs included a variety of host factors, including cytokines and proteins involved in copper metabolism and autophagy [171]. Interestingly, proinflammatory cytokines from macrophages and dendritic cells were suppressed by vaccine-induced EVs (e.g., miR-451a, miR-5100, or miR-7704) [172]. Although much of the current work has focused on single influenza virus strains, important strain specific EV dynamics have begun to be identified. In one study, nearly half of exosomal miRNAs were conserved between H1N1 and H7N7 infection in A549 cells [173]. Of the differentially expressed EVs, they were >10-fold during infection with the highly pathogenic H7N7 than with uninfected samples. A better understanding of these dynamics and temporal- and strain-specific differences could provide important insight into pathogenicity and pinpoint new therapeutic and universal influenza vaccine targets.

### 4.4. Hepatitis C Virus and EVs 

HCV belongs to a family of human virus called Flaviviridae characterized by positive-sense single-stranded RNA that encodes precursor polyprotein that is cleaved into three structural proteins comprising of core protein p22 with envelope glycoprotein E1 & E2, and seven non-structural proteins that play a role in viral pathogenesis [131,134]. The chronic viral infection leads to hepatic inflammation that is associated with increased production of pro-inflammatory cytokines and chemokines from liver residential immune cells and immune cells recruited to the liver [174]. EVs are observed as major modifiers of cellular crosstalk between HCV-infected hepatocytes & immune cells [174]. In HCV pathogenesis EVs act as a double edge power by: (1) delivering vireo-independent HCV RNA and (2) obtaining antiviral immune responses [174]. The cellular vesicular pathway is exploited by HCV to congregate and release viral particles. This happens by releasing vesicles containing envelope glycoprotein E1 & E2, entire HCV genome & viral particles. When the vesicles containing these components enter the target cells, this helps to establish infection [175].

In systemic alteration of an immune response, major regulators commonly known as specifically enriched micro RNAs (miRNAs) are delivered by EVs. These are loaded into EVs and are involved in post-transcriptional regulation of gene expression, which is known to be influential for HCV replication [176,177]. This confirms that EVs have peculiar miRNA expression isolated from the sera of chronic HCV patients. Exosomes derived from HCV infected cells are responsible for developing infection to other uninfected cells. These exosomes carried viral RNA in complex with miR-122, Ago2, and HSP90 that support virus replication [133]. EVs, isolated from sera of patients with acute or chronic HCV or interferon-stimulated macrophage cultures, mediate inhibitory effects on HCV replication [178]. In co-culture models, the immunoregulatory effects of EVs were assessed on the replication of HCV. Stimulation with type I & II Interferon N, which is a fast but short-lasting EV-derived antiviral, leads to the production of macrophages by secreting various cytokines resulting in innate immunity. Thus, HCV replication in macrophages derives EV-mediated long-lasting inhibitory effects [178]. EVs released by HCV infected cells contain viral RNA that might trigger plasmacytoid dendritic cells to produce IFNα [132]. 

### 4.5. Coronaviruses and EVs

The emergence of the life-threatening “atypical pneumonia” caused by severe acute respiratory syndrome coronavirus (SARS-CoV) in the early 21st century has led to renewed interest in coronaviruses [179]. Coronaviruses belong to the family of RNA viruses and possess the largest genome among them. Similar to other viruses, their genome contains essential genes encoded for open reading frames 1a and 1b (ORF1ab), and viral structural proteins, which are required for virus replication, transcription, and virus assembly [180]. A newly emerged coronavirus disease in 2019 (COVID-19) is caused by a novel severe acute respiratory syndrome coronavirus-2 (SARS-CoV-2). SARS-CoV-2 infection spread within a few months after the first outbreak reported in December 2019 in China, which later became a worldwide crisis. With high morbidity, the disease is often characterized by an atypical severe pulmonary pneumonia [181,182]. The novel SARS-CoV-2 is closely related to SARS-CoV-1 coronavirus responsible for the SARS outbreak that emerged in late 2002 in China. Its subsequent worldwide spread had caused 8096 cases and 774 deaths by July 2003 [183]. SARS-CoV-2 infections, which has already infected >18 million people and caused the death of ~700,000 people world-wide, are presently occurring and represent an ongoing threat to public health. 399 out of 1590 cases in China reported having at least one comorbidity [184]. The risk of serious adverse outcomes of COVID-19 is especially pronounced in patients with comorbidities such as hypertension, diabetes, kidney, and cardiovascular diseases [184,185]. 

SARS-CoV encodes four structural proteins; spike glycoprotein (S), nucleocapsid protein (N), membrane protein (M) & small envelope glycoprotein (E) & several nonstructural proteins of unknown functions [186]. SARS-CoV-2 spike (S) glycoprotein interacts with angiotensin-converting enzyme 2 (ACE-2), the same receptor used by SARS-CoV to enter the target cells, in particular lung alveolar epithelial cells [187]. It has been demonstrated that EVs released by SARS-CoV-2 infected lung epithelial cells contain viral RNA fragments that were subsequently detected in the cardiomyocytes, suggesting viral RNA transmission via EVs [188]. SARS-CoV-2 is a positive-stranded RNA virus in an envelope with a genome of 29,727 nucleotides [189].

The spike protein S of SARS-CoV-2 (SARS-S) facilitates the viral fusion that can be triggered following the fusion-mediated conformational changes in the target cell receptor that mediates the entry of the virus into the target cells. Once inside the cell, a virus may utilize the exosome secretion pathway to enhance its pathogenesis and viral spread [188]. To find a vaccine against SARS-CoV-2, researchers performed exosome-based research, where they constructed chimeric S protein of the SARS by replacing cytoplasmic and transmembrane domains of SARS-S with G protein of the vesicular stomatitis virus. This chimeric S-protein was readily expressed on the cell surface, allowed entry of pseudotyped retroviral vectors, and was incorporated into exosomes. Subsequently, chimeric S protein-containing exosomes have been tested as a novel protein for vaccine immunogenicity against SARS-COV in mouse models [135]. Recently, preclinical studies have uncovered a therapeutic role of MSC-derived secretome or EVs in lung regeneration [190], which could offer a new therapeutic approach in treating severe COVID-19 infection [191,192]. Intravenous transplantation of ACE2-negative mesenchymal stem cells (MSCs) promoted recovery of patients from severe COVID-19 [193], thus supporting the hypothesis that binding of SARS-S protein through ACE2 expressed on MSC-derived small EVs could limit the viral infection through competitively inhibit the binding of SARS-S to ACE2 expressed on alveolar type II cells [194].

### 4.6. Other Viral Infections and EVs 

Epstein-Barr virus (EBV) is one of the Herpes viruses that hijack its host EVs. EBV infected cells release EVs that contain EBV-coding/non-coding miRNAs and transfer it to uninfected cells including B lymphocytes and epithelial cells [83,195]. The transfer of EBV-coding miRNAs to B lymphocytes, especially the Akata-lymphoblastoid cell lines-derived EVs, causes inflammatory responses of monocytes/macrophages and induces severe lymphoproliferative disease (LPD) [195]. EBV viral reactivation was recently detected in co-cultured latently EBV-infected BL cells in response to the transfer of EVs that contain epithelium-specific miRNAs from oropharyngeal epithelial cells [83]. EBV-infected cells can transfer non-coding RNAs such as BART and BHRF1 miRNAs via EVs to the target cells. Upon entry, miRNAs can be directed to cellular sites of miRNA-mediated gene repression, causing repression of their target genes CXCL11 and LMP1 [136]. EBV–infected nasopharyngeal carcinoma cells release EVs containing Galectin-9 protein that interacts with the Tim3 membrane receptor and induces apoptosis in T cells [137]. Similarly, exosomes released by these cells convey the viral protein Latent Membrane Protein 1 (LMP1) that provoke intrinsic T-cell inhibitory activity and thus modulate immune response mechanisms [138].

Herpes simplex virus 1 (HSV-1) is another Herpes virus that hijacked its host EVs. HSV-1-infected cells release EVs with different components based on their stage in the infection cycle [49]. Early in the lytic cycle, HSV-1 proteins cause remodeling to EVs’ cargos, which in turn cause virion egress from infected cells to uninfected cells [49]. HSV-1 EVs contain coding and non-coding RNAs and more importantly immune components, such as the stimulator of interferon genes (STING) [196]. A recent study demonstrated that STING-containing EVs play an important role in inhibiting viral replication during the lytic cycle, as well as inhibiting viral gene expression during the latent stage [141]. Another recent report illustrated that miR-H28 and miR-H29 are being expressed late in the virus infection cycle and transferred to uninfected cells via EVs [140]; miRNA-28 induces the formation of gamma interferon (IFN-γ) which blocks viral replication in uninfected cells but not in infected cells [197]. IFN-γ loaded EVs maximize viral transmission between individuals by diminishing the spread from infected cells to uninfected cells [197]. A study reported that HSV-1 encoded glycoprotein B (gB) modulates the immune response by manipulating the MHC class II processing pathway by diverting Human Leukocyte Antigen–DR (HLA-DR) molecules into the exosome pathway [139].

An EV vaccine for the hepatitis B virus (HBV) is currently under investigation. As in most of the viruses, EVs carry HBV viral proteins such as large S, Core and P proteins which participate in viral replication [142]. They also play many roles in HBV infection; they are responsible for HBV replication, innate immune response during infection, a biomarker for its diagnosis, and development of a possible vaccine [198,199]. A recent study elucidated that unmodified EVs can be attractive coadjutants to hepatitis B recombinant antigen (HBsAg), because it triggers the healthy mice immune response due to an increased IFN-γ concentration and accelerates the production of IgG antibodies [200]. HepG2.2.15 cells with integrated HBV genome release EVs containing HBV-miR-3 which represses viral protein production and HBV replication [143]. Moreover, the study elucidated that Engineered EVs that are loaded with exosome-anchoring protein Nef mutant (Nefmut) and HBV core protein can induce cytotoxic T lymphocyte (CTL) immunization in animals for HBV infection [201]. 

On the one hand, EVs are responsible for infection transfer from one cell to another. On the other hand, EVs are also responsible for antiviral response initiation by inducing the uninfected cells’ immune response [197,202]. Due to their abilities to activate the innate and adaptive immune response, EVs can be the future pathway for the treatment of many viral infections. So far, viruses that impair their host immune response such as human T-lymphotropic virus (HTLV-1) only use their host’s EVs to use viral proteins such as gp61, Tax, and HBZ to increase cell-to-cell contact and promote a potential increase in viral infection [144]. HTLV-1 EVs were found to contain a protein called TAX that is implicated with the dysregulation of the recipient cells’ immune response [144,202]. Interestingly, there are viruses that not only hijack host EVs, but also boost the production of EVs such as in ZIKA virus (ZIKV). EVs released from ZIKV-infected (C6/36) cells carry viral RNA and ZIKV-E protein that can trigger monocyte activation to induce mRNA expression of TNF-α [145]. ZIKV-infected cells have incrementation in their neutral Sphingomyelinase (nSMase)-2/SMPD3 gene expression and activity, which provokes the production and excretion of EVs in neurons. Treatment of ZIKV requires the hindrance of EV production through the inhibition of SMPD3s in neurons to prevent further neuronal death and virus spreading [203]. 

## 5. EV-Based Antiviral and Antiretroviral Therapy

With the introduction of antiretroviral therapy (ART), the morbidity and mortality associated with HIV infection have drastically reduced [204]. However, due to the presence of latent reservoirs and inadequate drug concentration in the central nervous system (CNS), the virus continues to replicate and causes a wide range of CNS pathologies, including HIV-associated neurocognitive disorders (HAND) [205]. Therefore, new drug delivery systems that facilitate drug passage across the BBB to effectively suppress the virus in CNS, with minimal/tolerable neurotoxicity need to be developed. EVs can be used as a potential drug delivery system as they can cross the BBB [206,207] with less immunogenicity. Further, in preclinical studies, EV-based drug delivery platforms have been shown to carry therapeutic small molecules across the BBB to help alleviate multiple CNS diseases, including Parkinson’s disease and brain cancer [208,209,210]. EVs that can be used as a drug delivery platform are mainly derived from exosomes that linked to an endolysosomal pathway. Exosomes released from dendritic cells are considered vaccine candidates for immunotherapy in diseases such as cancer. These exosomes can be further taken up by dendritic cells leading to a presentation of MHC-I or peptide complexes [211,212,213]. ARVs can be loaded into EVs to deliver them across the BBB to achieve viral suppression in the CNS [214]. Since the autoclaved exosomes show intrinsic stability at a physiological temperature [215], sterile drug-loaded EVs can be formulated. Large scale production of EV drug formulation can be achieved using an endogenous drug-loading method that uses cells to release EVs with target drugs encapsulated in vitro. EVs with encapsulated drugs are capable of targeting the diseased cell or tissue, with targeting characteristics [110]. This inherent feature could be used to deliver drugs selectively to their intended targets while abrogating off-target side effects. 

Virus-targeting antiviral drugs can include protease inhibitors (PIs), integrase inhibitors, nucleoside and nucleotide reverse transcriptase inhibitors, and nonnucleoside reverse-transcriptase inhibitors. An EV-based drug delivery platform with either HIV PIs alone or in combination with ritonavir is used as a pharmaco-enhancer or second line of therapy for the treatment of HIV [214]. EVs can also be used as a vehicle for delivery of CRISPR-associated endonuclease (Cas9) and potentially as the guide RNA (gRNA) to target nucleotide sequences within viral genomes [216,217]. Another therapeutic use of EVs is vaccination against infectious diseases and viral infection. EV-mediated delivery of mRNA encoding pathogenic proteins required for viral infection might be a vaccine candidate that can induce T helper 1 (Th1)-type immune responses and cell-mediated immunity, without the need to attenuate and inactivate pathogenic viruses or bacteria [216,218]. For the ongoing pandemic of COVID-19, anti-HIV PIs, other PIs, or other antiviral and antibacterial drugs can either be encapsulated in EVs derived from various cell lines using endogenous loading technique or from the plasma of patients using exogenous loading method for personalized therapy [214]. Repurposing FDA-approved antiviral drugs using EVs could be a fast way to get tested through clinical trials. 

Although the clinical research done on EVs seem promising for therapeutic application, several factors must be considered before translating EVs into clinics. At present, available EV isolation methods, such as ultracentrifugation, density gradient centrifugation, precipitation, size exclusion chromatography, affinity, and novel microfluidic techniques are not sensitive enough to distinguish EVs subpopulation due to lack of specificity, physical and chemical biomarkers [219]; therefore, a high level of standardization is required to compare EV protocols and results used across different laboratories before the adoption of EV therapy to various clinical applications. Also, EVs’ pharmacokinetics, half-life, and plasma stability, as well as the interaction of encapsulated drugs with EV components, EV-targeting, and immune clearance of EVs, are other limitations that need to be overcome before realizing the clinical applications of EVs in drug delivery.

## 6. Conclusions 

**Figure 1:**
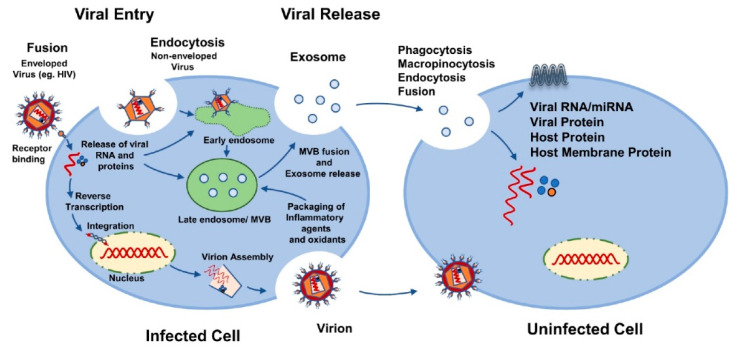
Possible mechanisms of viral spread using extracellular vesicles.

A growing body of evidence suggests that virus-infected cells produce EVs, encapsulated with viral proteins and parts of viral genetic material, and in some cases they carry the full infectious viral genome that facilitates viral infection and mediates immune responses (Figure 1). Notably, EVs can enhance viral infection by: (1) mediating transfer of chemokine co-receptors or cell surface proteins to null-target cells that do not express endogenous viral co-receptors; (2) helping viruses to evade the host immune system; (3) transferring of viral components (viral proteins and RNAs) to recipient cells, which induce cytotoxic effects on infected cells, leading to progressive loss of immune cells resulting from the apoptosis of uninfected bystander cells. Here, we aimed to shed light on how EVs potentially impact infection and the pathogenesis of various viruses. We also evaluated the potential utilization of EVs in antiviral and antiretroviral therapy, and in drug delivery. Characterizing EVs from virus-infected cells and their functional analyses could aid not only in the understanding of the mechanisms of viral infection but also in the utilization of EVs as a delivery system for therapeutic agents. 

## Figures and Tables

**Table 1:** Role of extracellular vesicles in viral pathogenesis and infection.

Virus	Type	Specific	Proposed Mechanism	Reference
HIV	Viral protein	Nef	HIV-infected cell-derived exosomes carrying negative regulatory factor (Nef) induces apoptosis in T-lymphocytes; Nef-transfected microglia-released Nef+exosomes reduce the blood–brain barrier (BBB) integrity	[112,114]
	Chemokines and receptors	CCR5, CXCR4, MCP-1	Facilitate the entry of HIV	[115]
	Proinflammatory markers	IL-6, TNF-β, IL-8	HIV-infected cells derived exosome containing TAR RNA plays a role in the increase of IL-6 and TNF-β in macrophages. HIV-infected U1 macrophages upon Cigarette smoke condensate (CSC) treatment enhanced the packaging of IL-6 in EVs; IL-8 served as a biomarker for HIV patients with altered immune function due to alcohol and tobacco abuse	[20,116,117]
	Host protein	APOBEC3G	Inhibit replication of viral infectivity factor (vif) -deficient and wild-type HIV-1 in recipient cells	[118]
	miRNA	vmiR-88 and vmiR-99	Triggers endosomal toll-like receptor (TLR) 8 and nuclear factor-κB (NF-κB) signaling, stimulating the release of TNFα by delivering EV to bystander macrophages, and may contribute to chronic immune activation	[119]
	Oxidative stress factors Cellular markers	CYP (1A1, 1B1, and 2A6), SOD1, CAT GFAP	Induce HIV replication. HIV-infected U1 macrophages upon CSC treatment promotes differential packaging of CYPs and AOEs in EVs Increased levels of glial fibrillary acidic protein (GFAP) in plasma EVs from HIV subjects can serve as a potential biomarker	[116,120,121]
HPV	mRNAs	E6 and E7	Contribute to viral immune-evasion and act in concert to promote tumor development through the interaction with multiple cellular proteins	[122,123]
	miRNA	miR-9, -20b, and let-7b	Cancer-associated, cellular pathways targeted by these miRNAs. Induce tumorigenesis through the effect of these microRNAs on their targets	[124]
		miR-222	Plays a role in cervical carcinogenesis, notably through the downregulation of p27 and phosphatase and tensin homolog deleted on chromosome 10 (PTEN)	[125]
		miR-7-5p	Favors cell proliferation	[126]
		miR-92a-3p	Possesses anti-apoptotic properties	[127]
	Proinflammatory mediators	CCL2 and TNFα	Inflammatory immune mediators	[24,124]
Influenza	Protein	Epithelial mucins MUC1, MUC4, and MUC16	Human airway-derived exosome-like vesicles containing mucins neutralize human influenza virus infection	[128]
	miRNA	miR-483-3p, hsa-miR-1975	Anti-viral and inflammatory response to influenza virus infection; suppresses influenza virus replication	[129,130]
HCV	Viral Genetic Material	RNA	Receptor independent viral transmission to hepatocytes; IFN-α production in plasmacytoid dendritic cells	[131,132]
	miRNA	miR-122	HCV transmission	[133]
	Envelope proteins	E1 and E2 glycoprotein	Modulate and transmit HCV infection	[134]
Coronavirus	Viral Protein	spike S proteins (SARS-CoV	Induce high levels of neutralizing antibodies, vaccine candidates for immunotherapy	[135]
**Other Viruses**				
EBV	miRNA	BHRF1 and BART miRNAs	miRNA-mediated repression of EBV target genes such as CXCL11 and LMP1	[136]
	Host protein	Galectin-9	This protein interacts with the Tim3 membrane receptor and induces apoptosis in T cells	[137]
	Viral protein	Latent Membrane Protein 1 (LMP-1)	Up-regulate adhesion molecules, such as ICAM-1, in recipient cells, promoting infectivity; modulate signaling pathway such as CD40 and FGF2	[138]
HSV	Viral Protein	viral glycoprotein B	Modulates immune responses to the viral antigen (Ag)	[139]
	Viral miRNAs	miR-H28, miR-H29	Restrict viral replication and cell-to-cell spread of viral infection	[140]
	Host protein	Stimulator of INF genes (STING) protein	Activates antiviral responses in recipient cells, Inhibits viral gene expression, and replication.	[141]
HBV	Viral proteins	large S, Core and P proteins	Hepatocytes secreted exosomes participate in virus replication	[142]
	Viral miRNAs	HBV-miR-3	Represses viral protein production and HBV replication	[143]
HTLV-1	Viral proteins	gp61, Tax, and HBZ	Increase cell-to-cell contact and promote a potential increase in viral spread	[144]
Zika	Viral genetic material and protein	RNA and ZIKV-E	EVs derived from Infected C6/36 cells promote infection and activation of monocytes with enhanced TNF-α mRNA expression.	[145]
